# Down syndrome frontal cortex layer III and layer V pyramidal neurons exhibit lamina specific degeneration in aged individuals

**DOI:** 10.1186/s40478-024-01891-z

**Published:** 2024-11-27

**Authors:** Melissa J. Alldred, Kyrillos W. Ibrahim, Harshitha Pidikiti, Gabriela Chiosis, Elliott J. Mufson, Grace E. Stutzmann, Stephen D. Ginsberg

**Affiliations:** 1 Center for Dementia Research, Nathan Kline Institute, 140 Old Orangeburg Road, Orangeburg, NY 10962, 845-398-2170 USA; 2 Department of Psychiatry, New York University Grossman School of Medicine, New York, NY USA; 3 Department of Neuroscience & Physiology, New York University Grossman School of Medicine, New York, NY USA; 4 NYU Neuroscience Institute, New York University Grossman School of Medicine, New York, NY USA; 5 Program in Chemical Biology, Sloan Kettering Institute, New York, NY USA; 6 Breast Cancer Medicine Service, Memorial Sloan Kettering Cancer Center, New York, NY USA; 7 Department of Translational Neuroscience and Neurology, Barrow Neurological Institute, Phoenix, AZ USA; 8 Center for Neurodegenerative Disease and Therapeutics, Rosalind Franklin University, The Chicago Medical School, North Chicago, IL USA

**Keywords:** Alzheimer’s disease, Bioinformatics, Down syndrome, Frontal cortex, Laser capture microdissection, RNA-sequencing, Selective vulnerability, Trisomy

## Abstract

Selective vulnerability of neuronal populations occurs in both Down syndrome (DS) and Alzheimer’s disease (AD), resulting in disproportional degeneration of pyramidal neurons (PNs) affecting memory and executive function. Elucidating the cellular mechanisms underlying the selective vulnerability of these populations will provide pivotal insights for disease progression in DS and AD. Single population RNA-sequencing analysis was performed on neurons critical for executive function, prefrontal cortex Brodmann area 9 (BA9) layer III (L3) and layer V (L5) excitatory PNs in postmortem human DS and age- and sex-matched control (CTR) brains. Data mining was performed on differentially expressed genes (DEGs) from PNs in each lamina with DEGs divergent between lamina identified and interrogated. Bioinformatic inquiry of L3 PNs revealed more unique/differentially expressed DEGs (uDEGs) than in L5 PNs in DS compared to CTR subjects, indicating gene dysregulation shows both spatial and cortical laminar projection neuron dependent dysregulation. DS triplicated human chromosome 21 (HSA21) comprised a subset of DEGs only dysregulated in L3 or L5 neurons, demonstrating partial cellular specificity in HSA21 expression. These HSA21 uDEGs had a disproportionally high number of noncoding RNAs, suggesting lamina specific dysfunctional gene regulation. L3 uDEGs revealed overall more dysregulation of cellular pathways and processes, many relevant to early AD pathogenesis, while L5 revealed processes suggestive of frank AD pathology. These findings indicate that trisomy differentially affects a subpopulation of uDEGs in L3 and L5 BA9 projection neurons in aged individuals with DS, which may inform circuit specific pathogenesis underlying DS and AD.

**Supplementary Information:**

The online version contains supplementary material available at 10.1186/s40478-024-01891-z.

## Introduction

Individuals with DS have intellectual disability, cognitive decline, and develop early onset AD pathology. These individuals represent the most prevalent trisomy to survive birth, with ~ 1 in 700 live births having triplication of HSA21 [65, 74]. Triplication of HSA21 causes deficits in learning, language, and memory acquisition and consolidation on a spectrum of severity [17, 22, 47, 64, 67], however, the exact molecular mechanisms underlying these deficits remain underexplored. Individuals with DS will develop AD pathology by their mid-30’s-40’s, including cortical thinning, brain atrophy, amyloid-beta peptide (Aβ) containing senile plaques followed by tau-positive neurofibrillary tangles (NFTs) [30, 48, 68, 98], with reports of amyloid deposition in individuals as early as their teens [59, 96]. While several trisomic genes have been implicated in AD pathology or memory impairments, including amyloid precursor protein (APP) [21, 23, 30, 78], dual specificity tyrosine phosphorylation regulated kinase 1 A (DYRK1A) [25, 43], transmembrane serine protease 2 (TMPRSS2) [2], and 4 of the 6 known genes encoding Type I interferon receptors [24, 28, 35, 87], little genomic information related to excitatory cortical projection neurons during the critical stages of pathogenesis is available. Elucidating the genomic dysregulation underlying selective vulnerability in neuronal pathogenesis remains a major understudied area. As such, characterizing differential gene expression in selectively vulnerable neurons during critical degenerative time periods in the aging DS population is an unmet need.

Cortical abnormalities initiate during DS fetal and postnatal development, including disorganized lamination patterning and reduced neuronal proliferation [18, 75, 91, 95]. Laminar disorganization is seen in all six cortical layers in multiple cortical regions [45, 91], including within prefrontal cortex [91]. An extensive delay in the appearance of PNs and interneurons occurs during early development in DS [91]. Throughout the lifespan there are aberrant laminar patterns, as well as abnormalities in brain volume, total grey matter, and white matter in adults with DS [18]. Examining the mechanistic consequences of abnormalities in cortical lamination remain a significant knowledge gap. Few ‘omics studies have been performed in human DS brains [32, 73], and little spatial information is available, often due to lack of postmortem DS tissue. Critical ‘omics studies, particularly profiling neurons selectively vulnerable to degeneration spatially and via single population analysis in DS and DS with AD pathology (DS + AD), are currently lacking.

Prefrontal cortex, including BA9, is critical for executive function integration, including attention, working memory, mood and motivation [14, 76]. Individuals with DS display impairments in executive function, including deficits in attention, working memory, and motor speed [83]. Executive function impairments positively correlate with a decline in cognition during aging in individuals with DS [13, 86]. Within the frontal cortex, L3 and L5 contain large pyramidal neurons that compose the cortico-cortical and cortico-thalamic connectomes (reviewed in [42, 46]). Further, excitatory neurons located in L3 and L5 display extensive NFT pathology in individuals with DS by the fourth decade of life [93, 96]. Although a single nucleus RNA sequencing (snRNA-seq) study found alterations in inhibitory to excitatory cell ratios and alterations in HSA21 genes in both excitatory and inhibitory neuronal subtypes in DS prefrontal cortex (BA8/BA9) [73], laminar differences were not reported. Whether prefrontal cortical projection neurons display similar transcriptome expression patterns and the accompanying mechanistic alterations independent of circuity during the onset and/or progression of cognitive decline in DS remains unknown.

Single population RNA sequencing (RNA-seq) is an unbiased ‘omics approach that enables precise interrogation of genes, canonical pathways, and extrapolating expression of encoded proteins. Laser capture microdissection (LCM) enables microisolation of individual identified cells that can be pooled for downstream analysis [37, 38] including neuronal subtypes from postmortem human brains and relevant animal models [4]. Recently, we performed single population RNA-seq analysis on LCM microisolated BA9 L3 and L5 PNs from aged individuals with DS + AD and CTR brains to interrogate gene expression changes that are convergently dysregulated (e.g., dysregulated in both PN populations concomitantly) [8]. We found ~ 5,296 DEGs in L3 (herein L3A) and ~ 4,329 DEGs for L5 (herein L5A) of which 2,329 DEGs were convergently dysregulated [8]. These DEGs showed significant upregulation of numerous HSA21 genes as well as dysregulation of thousands of DEGs not derived from HSA21, which were queried for mechanisms of dysregulation.

This single population study revealed downregulation of multiple pathways and processes involved in cognitive decline, including SNARE signaling, synaptic transmission, and spatial learning and memory along with upregulation of senescence and phosphorylation of proteins as well as many others [8], indicating DS + AD brains have genotype/disease specific alterations in excitatory cortical PNs. However, many DEGs were specifically dysregulated only in DS L3 or L5 PNs, which we term uDEGs. We hypothesize that uDEGs identify significant laminar based circuitry dependent expression differences, which we interrogate in the present report. We posit that the identification of uDEGs and relevant biological pathways will inform lamina specific gene expression alterations relevant to the selective vulnerability and laminar disorganization in the aged DS brain, which is also important for AD-related pathology and cognitive decline.

We performed a mining analysis of our single population low input RNA-seq dataset of L3 and L5 PNs obtained from BA9 via LCM from individuals with DS + AD compared to CTRs [8]. Here, we characterize uDEGs and examine lamina specific mechanistic pathways altered in human DS L3 and L5 PNs. Based on the degeneration profile, we postulate L3 PNs are more reactive to disease onset than L5 PNs. Further, we anticipate the unique pathways and processes identified utilizing uDEGs can be linked to functional changes during disease progression. Thus, in concert with the convergent gene expression study [8], divergent gene expression analysis may elucidate gene expression and pathway differences that are related to circuitry alterations present in DS + AD as well as degenerative phenotypes unique to each population of cortical projection neurons within L3 and L5 in DS.

## Materials and methods

### Single population RNA-seq

Gene expression data from GEO: GSE251939 was generated in the Ginsberg laboratory from DS (*n* = 12) and CTR (*n* = 17) L3 and L5 PNs isolated via LCM, with RNA isolated and RNA-seq library sequenced by New York University Grossman School of Medicine (NYUGSOM) Genome Technology Center (GTC) [8]. Briefly, BA9 brain tissue from DS and CTR subjects were matched by the following criteria: age, sex, postmortem interval (PMI), and when known race/ethnicity, and hemisphere. DS brain tissue displayed profound neuropathology for AD indicative of DS + AD as previously described [8], however antemortem cognitive status was not available. Frozen tissue blocks were cut at 20 μm on a cryostat and sections Nissl stained as described previously [8]. LCM was performed on ~ 500 PNs for each lamina (L3 and L5). RNA was isolated utilizing the miRNeasy micro kit (Qiagen, Germantown, MD), which isolates total RNA (mRNA, tRNA & rRNA) including short length micro-RNA (miRNA). RNA samples were split to perform two technical replicates and library preparation was performed utilizing the SMARTer Stranded Total RNA-Seq kit-Pico input Mammalian v3 (Takara Bio, Mountain View, CA). Sequencing was performed at the NYUGSOM GTC for paired end reads using NovaSeq 6000 (Illumina, San Diego, CA). Bioinformatics was performed on FastQ files for all samples with technical replicates merged to reduce variability as previously described [8]. Genes with over 0.1 counts per million (CPM) were analyzed using the DREAM pipeline with covariate analysis [49, 82]. DEGs at *p* < 0.05 were considered statistically significant [8].

### Pathway analyses

Pathway analyses were performed using Ingenuity Pathway Analysis (IPA; Qiagen) [57, 79], Kyoto Encyclopedia of Genes and Genomes (KEGG) [55], Gene Ontology (GO) [1, 12], and STRING [88] in Cytoscape (cutoff 0.4) [85]. Correlation and dot plots for DEGs were generated using ggplot package v3.4.2 in R. uDEGs for L3 and L5 PNs were analyzed by IPA, KEGG and GO, and the resultant significant pathways and processes were compared between lamina and also to all DEGs identified for L3 (L3A, 5,296 DEGs) and L5 (L5A, 4,390 DEGs) [8]. Graphpad Prism (v10.2.3) was utilized to generate heatmaps for IPA canonical pathways and disease and functions (D/Fs). STRING analysis in Cytoscape was performed on DS compared to CTR uDEGs for L3 and L5 PNs. DEGs that showed significant protein-protein interactions (PPIs) in the unique L3 and L5 datasets were filtered to isolate direct significant interactions and reanalyzed in STRING to determine key dysregulated targets. A Shiny package in R was utilized to build a web-based app to run GO and KEGG Analyses using R version 4.3.3 and RStudio version 2023.06.0 Build 421 [89]. This app was also utilized to filter keyword targets to identify classes of processes affected by genotype and lamina. R version 4.3.3 was used to create a script that separated GO processes unique to L3 PN uDEGs and unique to L5 PN uDEGs. Cohorts compared were L3 unique, L5 unique, L3A, L5A, and DEGs convergently dysregulated in both L3 and L5. Layer specific (LS) processes were then isolated. L3 LS and L5 LS represent those processes only significant in L3 or L5 uDEGs in conjunction with L3A or L5A, respectively. Gene percentages were calculated for each LS GO process, comparing the percentage of uDEGs to all DEGs for L3 or L5 PNs in each significantly dysregulated process. Percentage of uDEGs were averaged per bin using a script in R. Excel was utilized to generate pie and donut charts for GO processes. Ancestral plots for GO was performed using the online tool QuickGO [16].

## Results

### Differential gene expression within DS L3 and L5 PNs

**Fig. 1:**
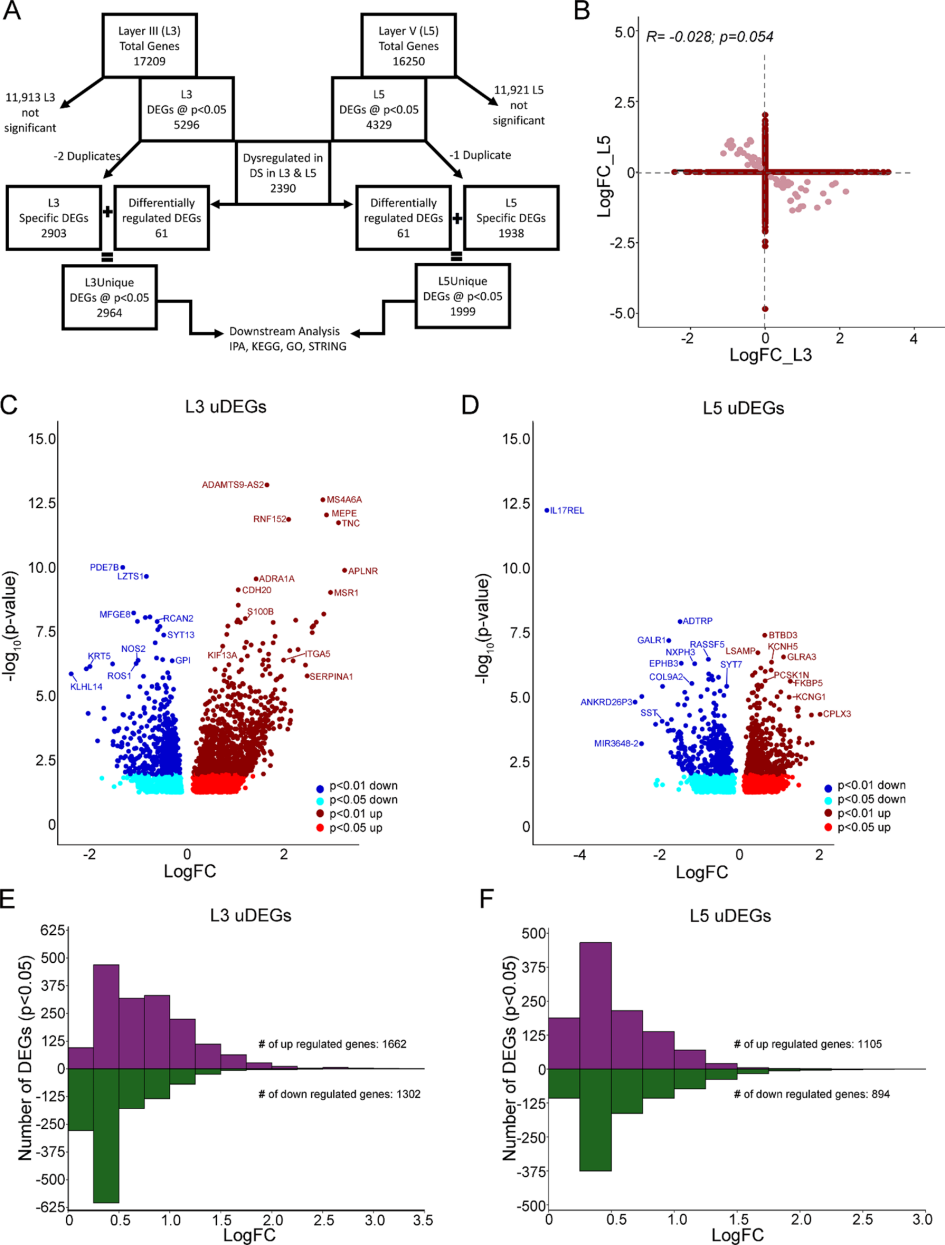
RNA-seq of BA9 L3 and L5 DS PNs exhibit unique and divergent gene expression. (**A**) Schematic representation of the L3 and L5 gene expression paradigm to isolate unique and divergently expressed genes in L3 or L5 PNs in DS compared to CTRs for downstream analysis. (**B**) Correlation plot shows dark red dots represent each DEG solely dysregulated in L3 or L5 shown as LFC. Light red dots represent divergent genes are upregulated in L5 and downregulated in L3 (top left quadrant) or vise-versa (bottom right quadrant). **C**, **D**. Volcano plots show L3 (**C**) or L5 (**D**) unique and divergent DEGs at a stringency of *p* < 0.01 (up, dark red; down, dark blue) and *p* < 0.05(up, red; down, light blue). **E**, **F**. Bar charts show L3 uDEGs (**E**) and L5 uDEGs (**F**) binned by LFC, indicating slightly more upregulated genes (purple) than downregulated (green) in DS compared to CTR cortical pyramidal neurons

The molecular signature of L3 and L5 PNs was interrogated to isolate unique and divergent gene expression changes in aged individuals with DS compared to age and sex-matched CTR subjects. A total of 5,296 DEGs were identified in L3 PNs (L3A; *p* < 0.05) and 4,329 DEGs were identified in L5 PNs (L5A; *p* < 0.05; Fig. 1A) [8]. To identify potential circuitry specific DEGs, L3 and L5 DEGs were compared with DEGs that were only significant in L3 (2,903; *p* < 0.05) or L5 (1,938; *p* < 0.05; Fig. 1A) and utilized for pathway analysis. Correlation analysis showed moderate association of L3A and L5A DEGs (Supplementary Fig. 1) [8]. However, uDEGs only significant in L3 or L5 PNs showed no significant correlation between lamina (dark red; Fig. 1B). Additionally, 61 divergent DEGs, which were upregulated in L3 and downregulated in L5 (or vice versa), were appended to L3 and L5 PN uDEGs (pink; Fig. 1B). Volcano plots demonstrate 2,964 upregulated and downregulated uDEGs were found in L3 PNs (Fig. 1C; Supplementary Table 1) and 1,999 uDEGs were found in L5 PNs (Fig. 1D; Supplementary Table 2). L3 uDEGs were binned by log fold change (LFC; 0.25 increments; Fig. 1E). Downregulated L3 PN uDEGs overall had smaller LFCs than upregulated uDEGs, with slightly more upregulated uDEGs overall. L5 PN uDEGs exhibited a similar ratio of upregulated and downregulated DEGs (Fig. 1F), and the LFC bins showed an equal distribution pattern with relatively more uDEGs with low LFCs.

**Fig. 2:**
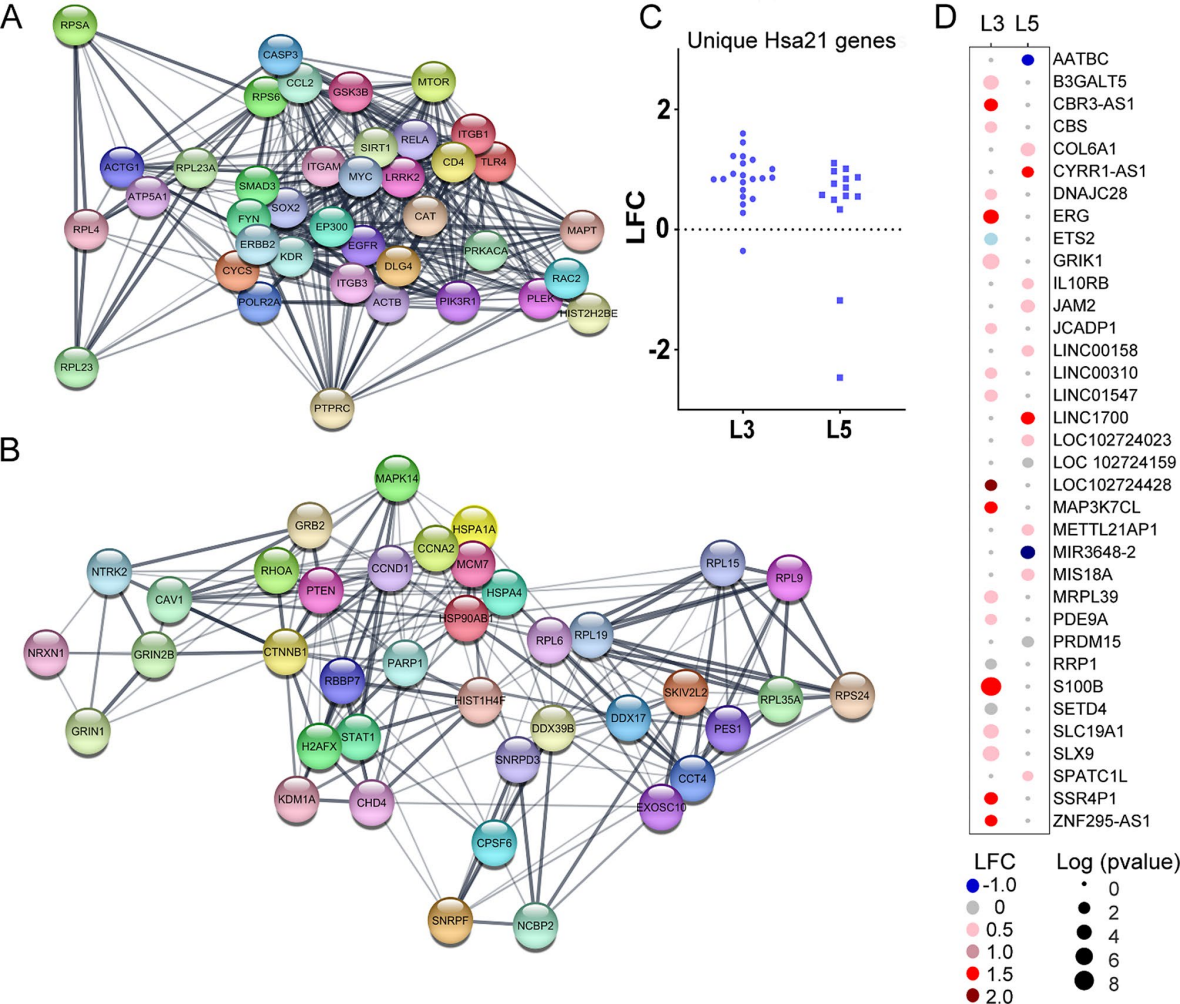
**A**. STRING analysis shows interactions of top L3 PN uDEGs in a PPI network indicating these genes have multiple interactions within the network. **B**. STRING analysis of L5 PNs showed fewer PPIs and more distinct groupings of the top interacting uDEGs. **C**. Scatter plot shows HSA21 triplicated uDEGs for L3 and L5 by LFC. **D**. Dot plot heatmap shows LFC (blue, downregulated; red, upregulated) of each significant uDEG for L3 and L5 PNs with Log (p-value) indicated by size of dot

STRING analysis in Cytoscape was performed on L3 PN uDEGs and revealed that of the 2,964 uDEGs, 2,644 uDEGs were identified in STRING for 23,143 total PPIs (Supplementary Table 3). PPIs were calculated in utilizing edge interactions from STRING for the uDEGs and the top 39 L3 PN uDEGs with at least 86 PPI partners were analyzed (Fig. 2A). Similarly, STRING analysis in Cytoscape was performed on L5 PN uDEGs and revealed of the 1,999 uDEGs, 1,759 uDEGs were identified in STRING for 9,035 total PPIs (Supplementary Table 4). The top 39 L5 PN uDEGs with at least 42 PPI partners were analyzed (Fig. 2B).

Several unique ribosomal proteins were identified in L3 and L5 PPI networks (Fig. 2A and B). L3 uDEGs had significant top hits with integrins including integrin subunit alpha M (ITGAM), integrin subunit beta 1 (ITGB1), and integrin subunit beta 3 (ITGB3), along with postsynaptic proteins including discs large MAGUK protein 4 (DLG4; Fig. 2A; Supplementary Table 3). L5 uDEGs had top hits for multiple heat-shock proteins including heat-shock protein 90 alpha family class b member 1 (HSP90AB1), heat-shock protein family 1 (Hsp70) member 1 A (HSPA1A), and heat-shock protein family 1 (Hsp70) member 4 (HSPA4), along with glutamate receptor subunits including glutamate ionotropic receptor N-methyl-D-aspartate (NMDA) type subunit 2B (GRIN2B; Fig. 2B; Supplementary Table 4).

### Lamina specific expression of HSA21 genes within BA9 L3 and L5 PNs

To determine the effect of HSA21 triplication on gene expression in BA9 L3 and L5 PNs, we queried all known HSA21 genes against the single population RNA-seq datasets derived for L3 and L5 neurons. Results indicated the majority of the expressed HSA21 genes showed convergent upregulation [8]. A small subset of HSA21 genes exhibited layer specific dysregulation, only significant in L3 (21 uDEGs) or L5 (14 uDEGs) in DS neurons compared to CTR subjects (Fig. 2C). No uDEGs were divergently expressed. All HSA21 uDEGs were uniquely dysregulated in either L3 or L5 determined by LFC and p-value (Fig. 2D). Interestingly, the L3 triplicated uDEGs included 6 non-coding RNAs (ncRNAs) and 2 ribosomal RNA (rRNA) genes, while the L5 uDEGs comprised 7 ncRNAs and 7 protein coding genes (Fig. 2D).

### Bioinformatic analysis reveals BA9 lamina specific vulnerabilities

**Fig. 3:**
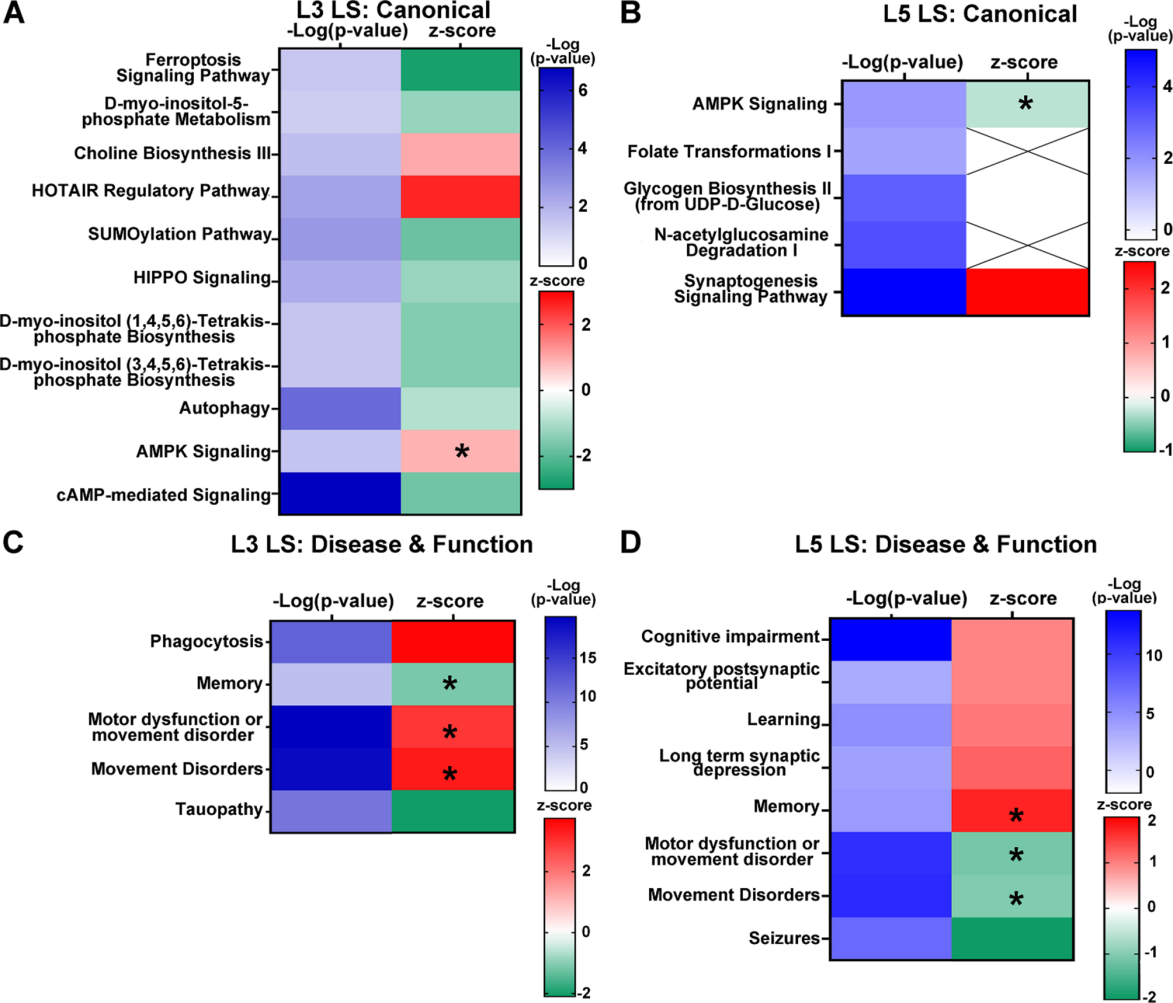
Lamina specific pathway changes were seen in DS L3 and L5 PNs. **A**. L3 uDEGs were analyzed by IPA and compared to L3A, L5 uDEG, and L5A IPA results. Select canonical pathways only dysregulated in L3 PNs are depicted using L3 uDEG -Log(p-value) and z-scores. **B**. L5 uDEGs were analyzed by IPA and compared to all groups as (**A**) with select L5 layer specific pathways shown. **C**. A subset of the L3 layer specific disease and functions indicated many D/Fs are divergently dysregulated in L3 and L5 (*). **D**. Select L5 layer specific disease and functions shown (*) marking divergent expression in L3 uDEGs. Key: -Log(p-value) shown in white-blue, while z-score is shown with upregulated pathways in red and downregulated pathways in green with white indicating a z-score of 0. Cross hatch in z-score indicates the IPA program cannot estimate regulatory status of this dysfunctional pathway

Bioinformatic inquiry was performed on L3 and L5 uDEGs from excitatory neurons, the results of which were compared to analysis of L3A and L5A DEGs. Differential pathway analysis was cross checked against L3/L5 convergent pathways and processes [8] to determine lamina specific dysregulation of DS PNs and significant dysregulation only seen examining uDEGs. IPA allowed for further analysis of directionality of pathways by the z-score rating, which indicates pathway dysregulation as upregulated (positive z-score), downregulated (negative z-score), and pathways in which directionality cannot be measured or has mixed effects (z-score either NA or zero) [57]. L3 uDEGs comprised select key canonical pathways that were dysregulated in L3A but were either *i*) not dysregulated in L5, or *ii*) divergently dysregulated in L5, referred to as L3 layer specific (L3 LS; Fig. 3A), including downregulation of several biosynthesis pathways and the SUMOylation pathway, and upregulation of choline biosynthesis III and AMPK Signaling (Fig. 3A). In contrast, IPA of L5 uDEGs and L5A indicated AMPK Signaling is downregulated in L5 PNs in a lamina specific manner (Fig. 3B). Further, L5 LS canonical pathways included glycogen biosynthesis II, which gave a 0 for z-score, while synaptogenesis signaling exhibited lamina specific upregulation (Fig. 3B). IPA D/F analysis revealed layer specific dysregulation of several key mechanisms. Memory and tauopathy D/Fs were downregulated by L3 LS gene expression, while the D/Fs of motor dysfunction and movement disorders, phagocytosis and movement disorders were upregulated (Fig. 3C). Conversely, the D/Fs memory, motor dysfunction and movement disorders, and movement disorders all showed divergent upregulation in L5 LS gene expression analysis (Fig. 3D). L5 showed significant LS upregulation of several D/Fs, including cognitive impairment, excitatory postsynaptic potential, and long-term synaptic depression (Fig. 3D). IPA data is available in Supplementary Tables (L3; Supplementary Tables 5–6 and L5; Supplementary Tables 7–8).

**Fig. 4:**
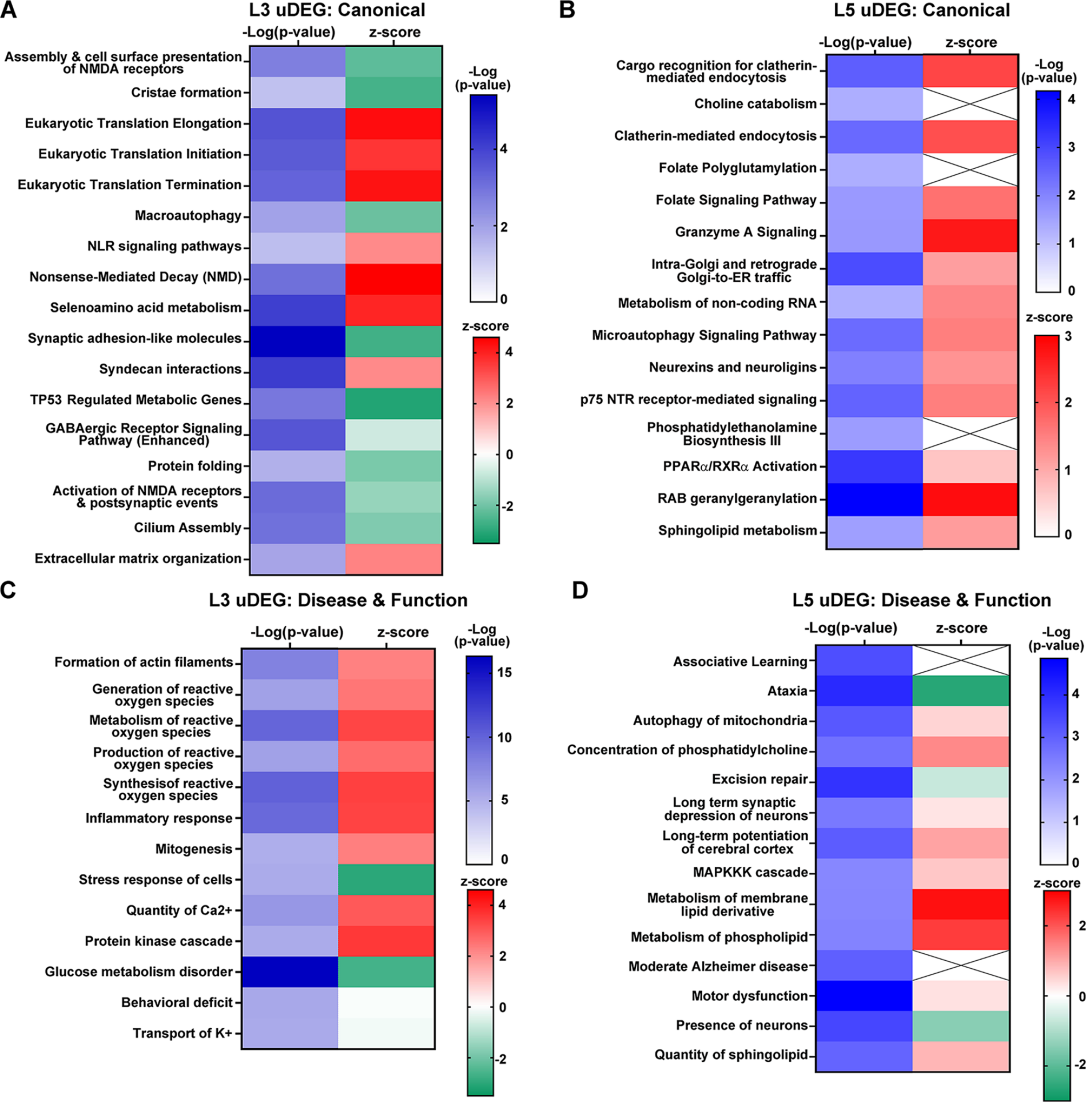
IPA pathway changes were seen with uDEGs from either DS L3 or L5 PNs. **A**. Select canonical pathways were identified as unique to L3 uDEGs by comparing IPA results from L3 uDEGs to IPA results from L3A DEGs, L5 uDEGs, and L5A DEGs. **B**. L5 uDEGs were analyzed by IPA and compared to all groups as in (**A**) with select L5 uDEG pathways shown. **C**. A subset of the L3 unique disease and functions are shown. **D**. Select L5 unique disease and functions are shown. Key: -Log(p-value) shown in white-blue, while z-score is shown with upregulated pathways in red and downregulated pathways in green. Cross hatch in z-score indicates the IPA program cannot estimate regulatory status of this dysfunctional pathway

Lamina specific pathway analysis showed several key circuitry specific gene expression findings. IPA revealed significant pathway dysregulation limited to L3 or L5 uDEGs. These pathways were not detected when examining L3A and L5A DEGs. This indicates genotype differences in DS may mask layer or circuit specific alterations in dysfunctional DS PNs. Examining L3 uDEGs revealed both upregulated and downregulated canonical pathways, including several specific to L3 PNs (Fig. 4A). Multiple intracellular functions appear significantly increased by L5 uDEGs, including clathrin-mediated endocytosis, neurexins and neuroligins, p75^NTR^ receptor mediated signaling, as well as RAB geranylgeranylation (Fig. 4B). IPA D/Fs showed significant hits exclusive to L3 uDEGs, including several key reactive oxygen species (ROS) mechanisms (Fig. 4C), while L5 uDEGs identified upregulated lipid metabolism, including metabolism of membrane lipid derivative and metabolism of phospholipid, with downregulation of excision repair and presence of neurons (Fig. 4D). Unique differences in DS L3 and L5 uDEGs suggests L3 and L5 PNs have distinct mechanisms of action that may reflect different stages of the degenerative process in the aged DS brain.

**Fig. 5:**
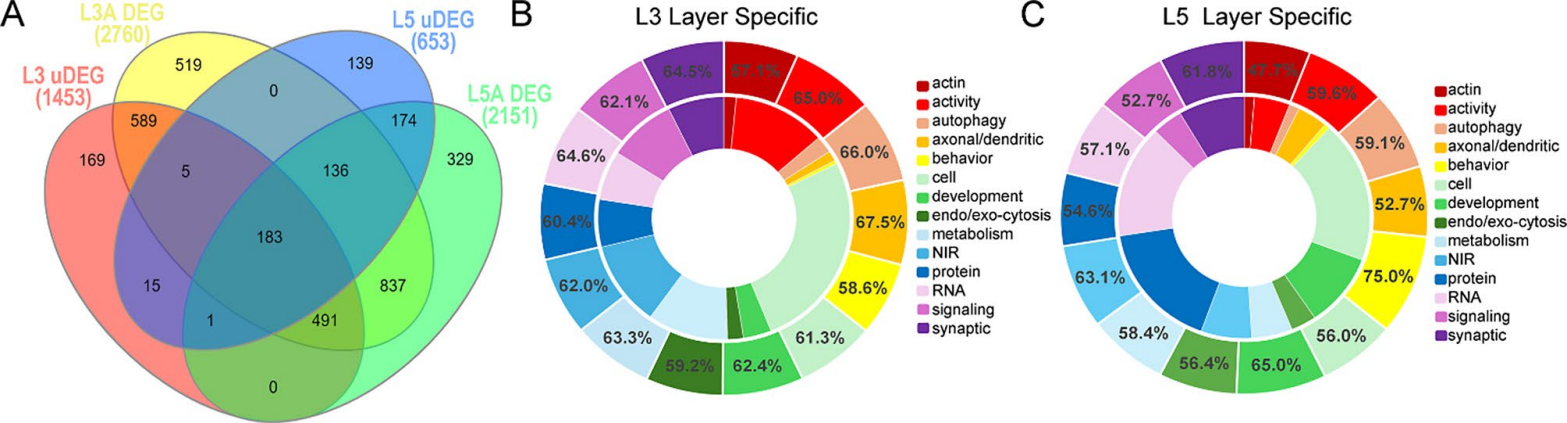
GO analysis indicates L3 and L5 PNs in DS show unique and lamina specific processes. **A**. Venn diagram indicates L3 uDEGs and L5 uDEGs represent a subset of the GO processes dysregulated in each layer, with L3 PNs having more dysregulated processes than L5 PNs. **B**. Donut chart shows comparison of L3 LS GO processes. The inner donut depicts the relative number of processes per bin and the outer donut is the average percentage of total DEGs from L3A compared to L3 uDEGs per process in each bin. **C**. Donut chart of L5 uDEG and all L5 DEG GO processes with the inner donut indicating relative amounts of dysregulated LS processes in each bin and the outer donut showing the mean percentage of uDEGs driving the processes within each bin

GO analysis was conducted to examine biological processes, molecular functions and cellular components to determine LS and unique processes dysregulated in uDEGs between laminae. GO analysis is available in Supplementary Tables 9–10. Analysis of lamina specific GO processes were examined with the same methodology as IPA. L3 PNs had 589 total LS processes, while L5 PNs had 174 LS processes (Fig. 5A). The total number of DEGs was compared to uDEGs for each LS process and represented as a percentage of contribution. Each process was then binned based on the uDEG percentage. L3 PNs had 47% of the processes with 60–70% uDEG contribution, followed by 38% of the processes with 50–60% uDEG contribution (Supplementary Fig. 2A). L5 PN GO processes had 44% of the processes with 50–60% uDEG contribution, followed by 25% of the processes with 60–70% uDEG contribution, and 20% of the processes had < 50% uDEGs (Supplementary Fig. 2B). LS processes were binned by category [8, 9]. In L3, the top categories were “axonal and dendritic”, which with 6 processes that averaged of 67.5% uDEG contribution, followed by “autophagy” with 10 processes that averaged 66% uDEGs, “activity” with 53 processes that averaged 65% uDEG contribution (Fig. 5B). L5 had different top categories with “behavior” that averaged 75% uDEG contribution, with only 1 dysregulated process (vocalization behavior, Supplementary Table 10), “development” with 15 processes that averaged 65% uDEG contribution, followed by “neurotransmitter, ion and receptor” (NIR) with 10 processes that averaged 63.1% uDEG contribution (Fig. 5C).

**Fig. 6:**
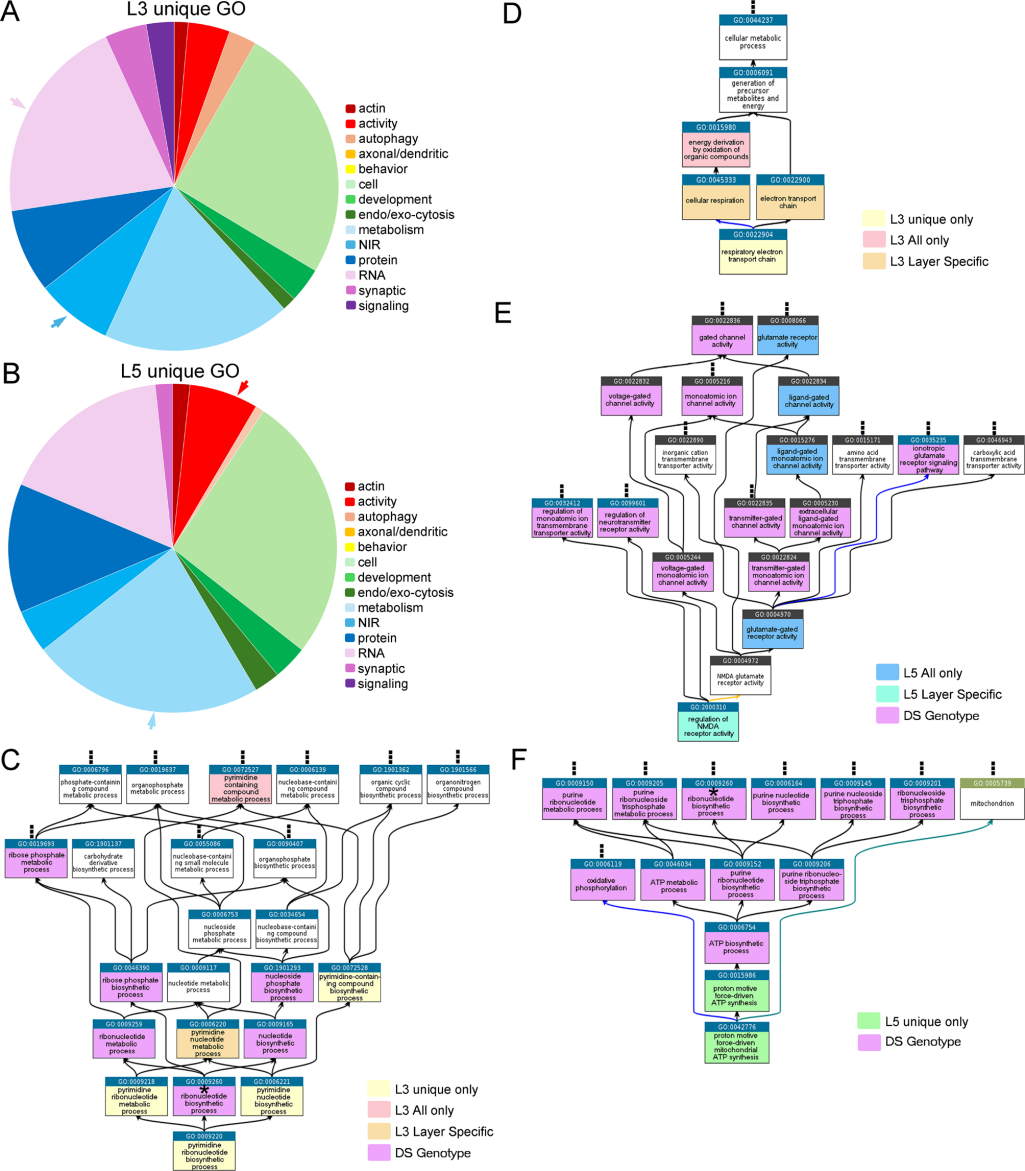
uDEGs were analyzed for uniquely dysregulated GO processes from L3 PNs and L5 PNs in individuals with DS compared to CTR subjects. **A.** Significant GO processes using L3 uDEGs show relatively more neurotransmitter, ion and receptor (NIR) and RNA processes (arrows) compared to L5 uDEGs. **B**. L5 uDEGs show relatively more metabolism and activity processes (arrows) compared to L3 uDEGs. **C**. The specific GO process 0009220, pyramidine ribonucleotide biosynthetic process, was dysregulated in L3 uDEGs via ancestral plots [16], which indicates the overall dysregulation of RNA metabolism. **D**. L3 uDEGs indicated the specific GO: 0022904, respiratory electron transport chain, is specifically dysregulated as part of the oxidative processes dysregulated by genotype. **E**. GO:2000310 regulation of NMDA receptor activity was dysregulated in L5 layer specifically, with ancestral plots showing upstream dysregulation is both specific to L5 and genotype specific to DS PNs. **F**. GO:0042776, proton motive force-driven mitochondrial ATP synthesis, a metabolic pathway, was uniquely dysregulated in L5 uDEGs, with ancestral plots showing L5 and genotype dysregulation of metabolic pathways. (Key: yellow = L3 uDEG; orange = L3 layer specific; green = L5 uDEG; cyan = L5 layer specific and purple = genotype changes seen in L3 and L5; * indicates a pathway seen in multiple ancestral plots)

A total of 169 GO processes were significant in L3 uDEGs, which were binned by category (Fig. 6A). L5 uDEGs displayed 139 significantly different GO processes (Fig. 6B). Binned categories were compared between L3 (Fig. 6A) and L5 (Fig. 6B) uDEGs, using the relative percentage of the total unique GO processes. L3 uDEGs had relatively more processes dysfunctional in the “RNA” and “NIR” categories, while L5 uDEGs had relatively more dysfunctional processes in “activity” and “metabolism” categories (arrows; Fig. 6A-B). Ancestral plots were generated on two L3 unique GO processes utilizing “Quick GO” [16]. Processes were queried against GO results from L3 and L5 uDEGs, and L3A and L5A. Ancestral plots for GO:0009220, pyrimidine ribonucleotide biosynthetic process (Fig. 6C) and GO:0022904, respiratory electron transport chain (Fig. 6D) linked key GO processes dysregulated throughout the hierarchy in an L3 specific (L3 unique = yellow; L3 LS = orange, L3A only = pink) or genotype specific manner (purple) for DS PNs compared to CTR PNs. Analysis of L5 specific processes using ancestral plots in “Quick GO” revealed GO:2000310 regulation of NMDA receptor activity (L5 LS = teal; Fig. 6E), with many of the upstream targets significantly dysregulated in L5A (blue) or by genotype (purple). GO:0042776, proton motive force-driven mitochondrial ATP synthesis was specifically dysregulated in L5 uDEGs (green), along with the upstream GO: 0015986, proton motive force-driven ATP synthesis. However, upstream ATP metabolism processes and oxidative phosphorylation were genotype specific to DS PNs (purple; Fig. 6F). GO analysis indicates L3 and L5 have both unique and convergent pathways dysregulated within DS PNs that may underlie specific connectome deficits and degenerative processes.

## Discussion

We postulate that prefrontal cortex L3 and L5 DS PN uDEGs underlie circuitry-based degeneration and reveal mechanistic pathway differences driven by lamina specific neuronal dysregulation in individuals with DS compared to age-matched CTR subjects. Interrogating postmortem BA9 PNs from L3 and L5 using single population RNA-seq elucidated thousands of DEGs in aged individuals with DS compared to CTR subjects [8]. To determine circuit specific alterations, we compared differential expression of L3 and L5 PN populations for unique and divergent gene expression. We identified uDEGs for each lamina examined, L3 and L5, with L3 PNs showing a significantly higher number of uDEGs. Bioinformatic inquiry of uDEGs in L3 and L5 PNs by STRING, IPA, and GO analysis revealed lamina specific canonical pathways, D/Fs, and mechanistic processes suggestive of circuitry specific dysregulation in DS PNs.

To determine the effect of HSA21 triplication on lamina specific gene expression, HSA21 DEGs were investigated in L3 and L5 DS PNs. A small subset of triplicated genes were uniquely dysregulated in either L3 or L5 PNs. Interestingly, many of the triplicated uDEGs were ncRNAs or rRNAs. Loc102724428 was originally identified as a ncRNA, however, more recent evidence shows Loc102724428 encodes salt inducible kinase 1 (SIK1). A recent report indicates SIK1 downregulates synaptic α-amino-3-hydroxy-5-methyl-4-isoxazolepropionic acid (AMPA) receptors and contributes to cognitive decline in AD [51]. Triplicated L3 PN uDEGs included 6 ncRNAs and 2 rRNAs. Many of these ncRNAs have few, if any publications, regarding their functionality in brain. However, ZNF295 antisense RNA1 (ZNF295-AS1) has been suggested to inhibit autophagy [80], which is specifically downregulated in L3 PNs (Fig. 4A). The mitochondrial ribosomal protein MRPL39 is involved in pediatric onset of mitochondrial disease [11] and is upregulated in L3 DS PNs (Fig. 2D). Linc01547 has been linked to autophagy in the tumor microenvironment [61]. Linc00310 shows increased expression in coronary artery disease [60], and increased expression of Linc00310 is a marker for breast cancer [63]. In L5 PNs, 50% (7/14, Fig. 2D) of the HSA21 uDEGs were ncRNAs. Although information on the functionality of these ncRNAs remains limited, it has been reported that Linc00158 overexpression inhibits apoptosis by promoting autophagy in spinal cord injury [81] and apoptosis associated transcript in bladder cancer (AATBC) stimulates mitochondrial function in adipocytes [44]. Further study of the function of ncRNAs is needed, especially in regard to ncRNAs dysregulated in DS PNs.

Lamina specific gene expression analysis showed L3 specific alterations in metabolism pathways including D-myo-inositol 5-phosphate metabolism and choline biosynthesis III, both involved in phospholipid metabolism [50, 58]. Previous studies have shown increased myo-inositol (mIns) and choline in the brain of an aging mouse model of AD [39] and in humans they negatively correlate with visuospatial working memory [62]. Increased mIns have been reported in individuals with DS [15], while choline levels are decreased in the young DS brain [34]. The downregulation of mIns metabolism and the upregulation of choline biosynthesis suggests onset of cellular metabolism dysfunction in L3 DS PNs.

In the present study, we also demonstrate that L3 PNs show prominent selective downregulation in autophagy and recycling pathways in aged DS + AD brains (Fig. 3A), which has been shown in the brain of DS mouse models, and in human postmortem DS and AD brain tissues [19–21, 52, 56, 69, 72]. When examining L3 LS D/Fs, we noted phagocytosis was upregulated (Fig. 3C), along with downregulation of the ferroptosis signaling pathway (Fig. 3A). These findings are interesting in light of recent reports suggesting novel forms of cell death, beyond apoptosis in AD including phagocytosis and ferroptosis, play a role in disease pathogenesis and progression [66]. Studies indicate increased neuronal death caused by ferroptosis signaling in AD [94], contrary to the RNA-seq findings derived from L3 PNs that show a LS decrease in ferroptosis. However, like the present RNA-seq findings in DS, recent studies have shown increased phagocytosis in cognitively normal individuals with AD neuropathology [40, 54], suggesting upregulated phagocytosis may be a compensatory mechanism. Although immunohistochemical analysis of L3 PNs would inform on autophagy and phagocytosis status, lack of tissue accession has made these investigations beyond the scope of the present study. The spatial transcriptomic results presented herein suggest L3 PNs exhibit a degenerative profile that is consistent with the onset of AD and confirms L3 excitatory PNs are vulnerable to pathological AD degeneration.

Circuit specific dysregulation was further analyzed utilizing uDEGs and pathway analysis. Results demonstrate L3 PN specific upregulation of RNA translation, including eukaryotic translation initiation, elongation and termination. By contrast, downregulation was seen in activation of NMDA receptors and postsynaptic events and the GABAergic receptor signaling pathway. D/F analysis in IPA showed significant upregulation of ROS generation, metabolism, production and synthesis along with inflammatory response and formation of actin filaments. This suggests L3 PNs specifically have increased neuroinflammation and ROS dysregulation, combined with decreased functionality of receptor signaling. Neuroinflammation is strongly associated with AD neuropathology and is thought to occur at an early timepoint during disease progression [26, 33]. Mitochondrial fragmentation resulting from hyperglycemia, caused by two common risk factors for AD, type II diabetes mellitus and/or obesity, is driven by increased ROS formation [97]. Upregulation of ROS generation and the resulting mitochondrial dysfunction has been causally linked to age-associated cognitive decline and decreased synaptic activity, which is postulated to contribute to disease mechanisms prior to Aβ and tau pathology [90]. The downregulation of receptor activity suggests neuronal dysfunction, which aligns with the DS + AD phenotype suggesting these aged individuals with DS have cognitive impairment indicative of dementia onset.

In contrast to L3, L5 PNs display downregulation of AMP activated protein kinase (AMPK) signaling and increased synaptogenesis signaling (Fig. 3B). AMPK works as a glucose sensor and regulates energy homeostasis [70]. In the brain, AMPK protects against oxidative and metabolic insults in developing rodent hippocampal neurons [27]. AMPK activation has also been shown to promote autophagy dependent degradation of Aβ peptides in vitro [92]. Longitudinal studies of metformin, a type II diabetes mellitus therapeutic, which activates AMPK, have shown significant benefits on cognitive status although long term use was less effective [71]. Together, these studies suggest downregulation of AMPK decreases degradation of Aβ peptides and reduces protection against oxidative damage, indicating L5 PNs are being driven toward degeneration in DS. However, other mechanistic pathways indicate L5 neurons respond to these pathological insults with putative compensatory mechanisms, including increased synaptogenesis. Reactive synaptogenesis could result in an influx of new synaptic connections actively forming in the L5 neurons. Interestingly, increased adult neurogenesis is thought to be compensatory in AD [10], and increased expression of neurogenesis markers are found in postmortem hippocampal AD tissue [53]. Upregulation of memory and downregulation of movement disorders and seizures in D/Fs (Fig. 3D) correlates with increased synaptogenesis in DS L5 PNs, suggesting that L5 PNs are actively undergoing degeneration likely in response to AD pathogenesis.

IPA analysis revealed activation of the D/Fs including memory and motor dysfunction or movement disorders. Both memory and motor dysfunction or movement disorders showed lamina specific functional divergence, with memory presenting as downregulated in L3 uDEGs and upregulated in L5 uDEGs, while the opposite was true with motor dysfunction or movement disorders (Fig. 3C, D). Herein, L5 PN uDEGs exhibit an increased activation score for memory and a decreased activation score for motor dysfunction or movement disorders, which may be linked to the L5 PN subcortical connectivity. These findings lend support to previous work suggesting a primary role of L5 PNs in both motor function and cognition [77].

Commensurate with IPA analysis, GO analysis resulted in L3 PNs exhibiting significantly more uDEG GO processes compared to L5 PNs (Fig. 4A 5A 1453 versus 653), which correlates with the overall higher percentage of uDEGs driving L3 (Fig. 4B) compared to L5 (Fig. 4D) LS processes. The top percentages of uDEGs were binned to “activity”, “autophagy”. and “metabolism” for L3 uDEGs, which correlates with IPA findings. When examining unique processes, L3 uDEGs showed a higher percentage of processes in “NIR” and “RNA”. Evaluation of RNA processes found that many processes specifically dysregulated in L3 PNs were involved in RNA biosynthesis (Fig. 6C), while others were ubiquitous to L3 and L5 DS PNs compared to CTR subjects. Dysregulation of RNA processing, including transcription, splicing and translation, have been seen by high throughput proteomics in AD along with functional differences of RNA binding proteins [84], which are postulated to play a significant role in early preclinical AD progression. The observation that L3 PNs show unique dysregulation of metabolism of RNA suggests that RNA transcription and modification are specifically disrupted in these neurons in individuals with DS.

GO analysis of L5 PN uDEGs showed signs of signaling dysfunction, with multiple processes of glutamate receptor activity dysregulated including regulation of NMDA receptor activity (Fig. 6D). Hyperexcitability is a hallmark of DS [29], along with alterations in long term synaptic depression and long-term potentiation [41], both of which were upregulated in L5 uDEGs by IPA (Fig. 4D). While “metabolism” was disrupted in both L3 and L5 PNs, the mechanism underlying this dysfunction showed unique processes dysregulated in L3 (Fig. 6C) and L5 PNs (Fig. 6F). Previously, we have shown in a mouse model of DS, basal forebrain cholinergic neurons have highly significant oxidative phosphorylation alterations [5–7], which were mimicked by GO processes involved in cellular respiration within L3 and L5 PNs. L3 PNs were dysregulated in the respiratory electron transport chain (GO: 0022904) and electron transport chain (GO: 0022900; Fig. 6E). In contrast, L5 PNs show dysregulation of 2 proton motive force-driven ATP synthesis (GO: 0042776 and GO: 0015986, Fig. 6F), which are part of the ATP biosynthetic (GO: 0006754) and oxidative phosphorylation (GO: 0006119) processes, dysregulated within both L3 and L5 PNs in DS. This suggests, while cellular metabolism is disrupted in DS, the mechanisms of action may be unique to each lamina based on cortico-cortical and cortico-thalamic connections.

Study caveats include validation of key targets that are cell-type or circuitry-specific, which is technically impractical, due to the amount of RNA or protein needed from single population LCM acquired neurons. Previous studies indicate RNA-seq results are replicated by RT-qPCR and protein analysis [5, 7–9, 31, 36], but these data are derived from lamina specific neuronal samples. Additional studies are required to determine how gene expression compares to proteomic differences in single cell and single population studies in postmortem DS cortex. The age range for the DS cohort was large, no cases lacked AD pathology, nor were younger DS cases prior to the development of AD pathology available to differentiate between DS and DS + AD. Access to younger postmortem DS brains will allow the differentiation of gene expression alterations due to trisomy compared to AD. The recently founded Down syndrome Biobank Consortium (DSBC) [3] will aid in tissue accession of cognitively evaluated cases at all ages, and sample distribution for future studies. However, at the time of this study no additional frozen tissue samples or subsequent single population RNA-seq reads were available. Planned future studies include additional brain regions and cell types (e.g., GABAergic interneurons) to determine regional and circuit specificity in aged DS brains in regions vulnerable to degeneration during AD onset and/or progression.

## Conclusions

BA9 L3 and L5 PNs display uDEGs, which may underlie neuronal circuit specific alterations in the DS + AD brain. Bioinformatic analysis indicates that DS L3 PNs have approximately 30% more dysregulated genes compared to L5 PNs, which resulted in more dysregulated pathways and processes in L3 PNs. RNA expression and metabolism as well as autophagy related pathways in L3 PNs were highlighted as uniquely dysregulated or functionally divergent between laminae. We postulate L3 PNs undergo early onset pathogenesis linked to preclinical and/or early stages of AD. In contrast, data from L5 PNs suggest that these neurons are more advanced in the degenerative process. We also observed HSA21 triplicated DEGs specifically dysregulated only in L3 or L5 PNs, with about half of these uDEGs being ncRNAs, suggesting that these regulatory elements are circuitry specific modulators of gene expression. Taken in the context of laminar convergence of DEGs [8], we postulate that uDEGs and pathways specifically dysregulated in L3 or L5 BA9 neurons elucidate key targets for the treatment of vulnerability in individuals with DS that also informs AD onset and progression.

## Electronic supplementary material

Below is the link to the electronic supplementary material.

**Supplementary Material 1:** Supplementary Figure 1. DEGs from L3 and L5 PNs in individuals with DS compared to CTR subjects were correlated by LFC in L5 PNs (y-axis) and L3 PNs (x-axis). Black dots represent DEGs convergently dysregulated, while pink dots represent divergent DEGs and dark red dots indicate DEGs that are only significantly dysregulated in L5 (along dashed 0 y-axis) or L3 (along dashed 0 x-axis), indicating that they show no or low non-significant dysregulation in L3 or L5 respectively. * Figure adapted from [8].

**Supplementary Material 2:** Supplementary Figure 2. A. Pie chart shows comparison of the relative percentage of DEGs from L3 unique compared to L3A for all 589 of the lamina specific GO processes B. Pie chart shows the relative percentage of DEGs in the 139 L5 LS GO processes comparing L5 unique to all L5, indicating L5 uDEGs account for a lower percentage of the DEGs driving the dysregulated GO processes.

Supplementary Material 3

Supplementary Material 4

Supplementary Material 5

Supplementary Material 6

Supplementary Material 7

Supplementary Material 8

Supplementary Material 9

Supplementary Material 10

Supplementary Material 11

Supplementary Material 12

## Data Availability

The dataset(s) supporting the conclusions of this article are available as follows. RNA-seq data analyzed within this study are available from GEO (www.ncbi.nlm.nih.gov/geo; GSE251939) or from the corresponding author upon request. LCM images for brain tissue (syn55354925) and RNA/library metadata (syn570405238 and syn61668772) are available from synapse.org upon request.

## Abbreviations

AATBC: Apoptosis associated transcript in bladder cancer
Aβ: Amyloid-beta peptide
AD: Alzheimer’s disease
APP: Amyloid-beta precursor protein
AMPA: α-amino-3-hydroxy-5-methyl-4-isoxazolepropionic acid
AMPK: AMP activated protein kinase
BA9: Brodmann area 9
CPM: Counts per million
CTR: Control
DEG: Differentially expressed gene
D/Fs: Disease and functions
DLG4: Discs large MAGUK protein 4
DS: Down syndrome
DS + AD: Down syndrome with Alzheimer’s disease pathology
DYRK1A: Dual specificity tyrosine phosphorylation regulated kinase 1 A
GO: Gene Ontology
GRIN2B: Glutamate ionotropic receptor NMDA type subunit 2B
GTC: Genome Technology Center
HSA21: Human chromosome 21
HSP90AB1: Heat-shock protein 90 alpha family class b member 1
HSPA1A: Heat-shock protein family 1 (Hsp70) member 1 A
HSPA4: Heat-shock protein family 1 (Hsp70) member 4 (HSPA4)
IPA: Ingenuity Pathway Analysis
ITGAM: Integrin subunit alpha M
ITGB1: Integrin subunit beta 1
ITBG3: Integrin subunit beta 3
KEGG: Kyoto Encyclopedia of Genes and Genomes
L3: Layer III
L5: Layer V
L3A: Layer III all
L5A: Layer V all
LCM: Laser capture microdissection
LFC: Log fold change
LS: Layer specific
mIns: Myo-inositol
MRPL39: Mitochondrial ribosomal protein L39
ncRNA: Non-coding RNA
NIR: Neurotransmitter, ion and receptor
NFTs: Neurofibrillary tangles
NMDA: N-methyl-D-aspartate
NYUGSOM: New York University Grossman School of Medicine
PMI: Postmortem interval
PN: Pyramidal neurons
PPIs: Protein-protein interactions
RNA-seq: RNA sequencing
ROS: Reactive oxygen species
rRNA: Ribosomal RNA
RT-qPCR: Reverse transcription quantitative polymerase chain reaction
snRNA-seq: Single nucleus RNA sequencing
SIK1: Salt inducible kinase 1 (aka Loc102724428)
TMPRSS2: Transmembrane serine protease 2
uDEG: Unique differentially expressed gene
ZNF295: AS1-ZNF295 antisense RNA1
