# Supplementary figures and images for: Down syndrome frontal cortex layer III and layer V pyramidal neurons exhibit lamina specific degeneration in aged individuals
Source: Acta Neuropathol Commun. 2024 Nov 27;12:182. doi: 10.1186/s40478-024-01891-z (PMC11603868; doi:10.1186/s40478-024-01891-z)

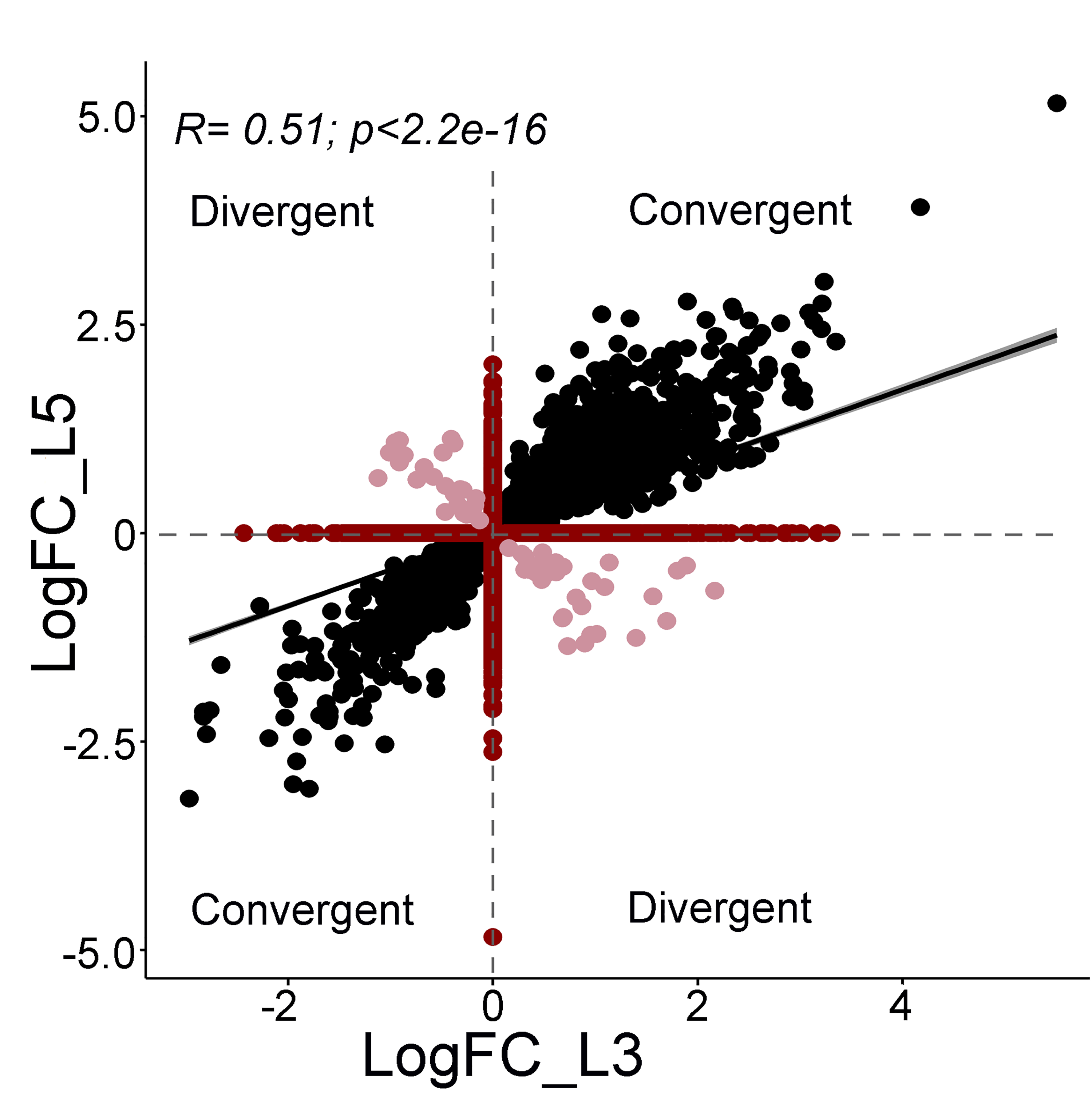

Supplement: Supplementary file 1 — Supplementary Material 1: Supplementary Figure 1. DEGs from L3 and L5 PNs in individuals with DS compared to CTR subjects were correlated by LFC in L5 PNs (y-axis) and L3 PNs (x-axis). Black dots represent DEGs convergently dysregulated, while pink dots represent divergent DEGs and dark red dots indicate DEGs that are only significantly dysregulated in L5 (along dashed 0 y-axis) or L3 (along dashed 0 x-axis), indicating that they show no or low non-significant dysregulation in L3 or L5 respectively. * Figure adapted from [8]. [file 40478_2024_1891_MOESM1_ESM.tif]

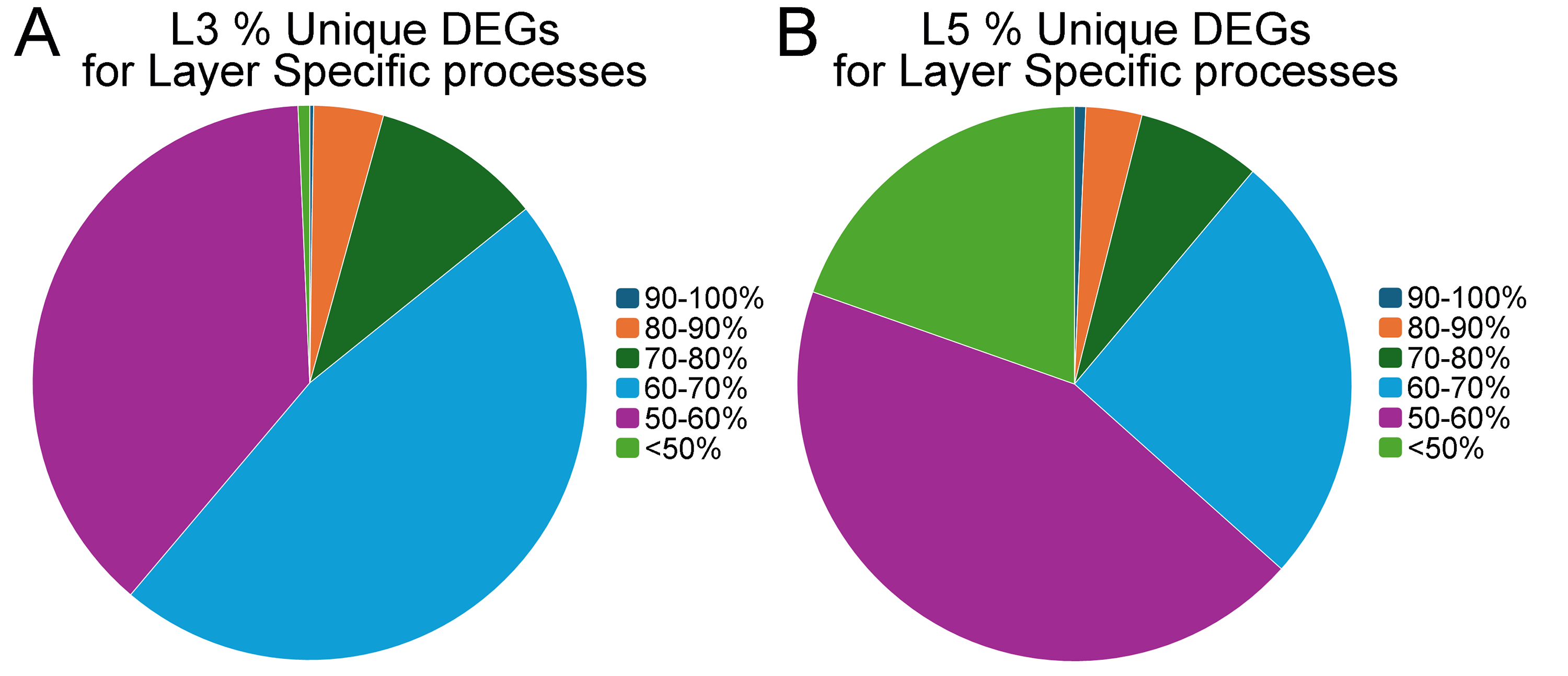

Supplement: Supplementary file 2 — Supplementary Material 2: Supplementary Figure 2. A. Pie chart shows comparison of the relative percentage of DEGs from L3 unique compared to L3A for all 589 of the lamina specific GO processes B. Pie chart shows the relative percentage of DEGs in the 139 L5 LS GO processes comparing L5 unique to all L5, indicating L5 uDEGs account for a lower percentage of the DEGs driving the dysregulated GO processes. [file 40478_2024_1891_MOESM2_ESM.tif]
